# Dominance of DENV‐1 and Flavivirus Serological Cross‐Reactivity During the 2016 Dengue Outbreak in Vietnam

**DOI:** 10.1002/jmv.70569

**Published:** 2025-08-21

**Authors:** Do Duc Anh, Do Huy Loc, Hoang Van Tong, Do Thi Huyen Dieu, Ngo Thu Hang, Nguyen Huu Lanh, Nguyen Thi Hong Nhung, Peter G. Kremsner, Le Huu Song, Nguyen Linh Toan, Thirumalaisamy P. Velavan

**Affiliations:** ^1^ Institute of Tropical Medicine University of Tübingen and German Center for Infection Research (DZIF) Tübingen Germany; ^2^ Vietnamese ‐ German Center for Medical Research (VG‐CARE) Hanoi Vietnam; ^3^ Department Pathophysiology Vietnam Military Medical University Hanoi Vietnam; ^4^ Binh Dinh Medical College Qui Nhon Gia Lai Vietnam; ^5^ Binh Dinh Hospital Qui Nhon Gia Lai Vietnam; ^6^ Centre de Recherches Médicales de Lambaréné (CERMEL) Lambarene Gabon; ^7^ 108 Military Central Hospital Hanoi Vietnam; ^8^ Faculty of Medicine Duy Tan University Da Nang Vietnam

**Keywords:** cross‐reactivity, dengue, dengue genotypes, flavivirus, Vietnam

## Abstract

Flaviviruses such as dengue virus (DENV), Zika virus (ZIKV), and Japanese encephalitis virus (JEV) pose a major health burden in Vietnam, where overlapping clinical features and serological cross‐reactivity complicate accurate diagnosis and outbreak control. This study aimed to investigate circulating DENV serotypes and assess serological cross‐reactivity with other flaviviruses during the 2016 dengue outbreak in central Vietnam. Aretrospective study was conducted on 146 hospitalized dengue patients during the 2016 outbreak in Binh Dinh province. Laboratory diagnosis included NS1antigen testing, ELISA (IgM/IgG), and real‐time RT‐PCR for DENV serotyping. IgM and IgG cross‐reactivity with five flaviviruses, including DENV, ZIKV, JEV, West Nile virus (WNV), and tick‐borne encephalitis virus (TBEV), and onealphavirus, chikungunya virus (CHIKV), was evaluated using ELISA. DENV‐1 positive samples were further analysed by sequencing the capsid‐premembrane (CprM) gene. DENV‐1 was the predominant serotype (86%), with all sequenced strains clustering within genotype I. Secondary infections were more frequent (64%) than primary infections (36%) and were associated with a significantly higher median age (*p* = 0.003) and elevated hs‐CRP levels (*p* = 0.029). Strong IgG cross‐reactivity was observed among flaviviruses, particularly DENV, JEV, WNV, and TBEV (*r* > 0.85), while ZIKV and CHIKV showed low seropositivity. Incontrast, IgM responses demonstrated greater virus specificity. Ten PCR‐negative cases showed broad serological reactivity, suggesting possible misdiagnosis or late‐stage infection. Our findings reveal that DENV‐1 genotype I was the predominant serotype during the 2016 outbreak in central Vietnam. Extensive IgG cross‐reactivity among flaviviruses hinders serological diagnosis, highlighting the need for integrated molecular surveillance to ensure accurate outbreak response.

## 1 Introduction

Flaviviruses, primarily transmitted by mosquitoes and ticks, present a significant public health challenge, particularly in tropical regions [1]. These viruses are responsible for a wide spectrum of clinical manifestations, ranging from mild febrile illness to severe, life‐threatening complications such as hemorrhagic fever, encephalitis, and shock syndromes [2, 3]. The rapid spread of flaviviruses, combined with overlapping symptoms and limited diagnostic capacity in many endemic regions, further complicates timely detection, clinical management, and outbreak control [4].

In Vietnam, dengue virus (DENV) is endemic and responsible for recurrent outbreaks of viral hemorrhagic fever. The risk of transmission increases during and after the rainy season, when populations of *Aedes* mosquitoes, the primary vectors of DENV, surge [5]. Between 2000 and 2020, Vietnam recorded an annual average of approximately 95 000 dengue cases, with a peak of 294 707 cases in 2019 [6]. In 2022, dengue cases surged dramatically, showing a fivefold increase compared to 2021, highlighting the increased burden on local healthcare systems during outbreak periods [7]. The burden and intensity of dengue outbreaks are determined by a complex interplay of virological, ecological, immunological, and socio‐environmental factors, including the co‐circulation of all four dengue virus serotypes (DENV‐1−DENV‐4) in Vietnam [8]. Notably, the emergence or reintroduction of new DENV serotypes or genotypes has been associated with outbreak intensity in endemic regions [9].

Variations in immunogenicity among the serotypes have also been associated with differences in disease severity [10]. For instance, DENV‐1 and DENV‐2 have been associated with more severe clinical outcomes in our previous study [7, 11]. Moreover, genetic diversity within each serotype may contribute to variability in disease severity, transmissibility, and immune response among infected individuals [12, 13]. Subtypes within DENV serotypes have also been reported to exhibit increased infectivity and virulence [14].

In addition, several other flaviviruses, including Zika virus (ZIKV), Japanese encephalitis virus (JEV), tick‐borne encephalitis virus (TBEV), and West Nile virus (WNV), have been documented in human and animal reservoirs in Vietnam [15, 16, 17, 18]. These flaviviruses can circulate silently in the population and occasionally present with unspecific clinical symptoms that overlap with dengue, complicating clinical diagnosis [16]. A further layer of complexity in clinical management arises from serological cross‐reactivity, which can lead to misdiagnosis in serological assays, particularly among closely related flaviviruses. This group of pathogens can also modulate immune responses through mechanisms such as antibody‐dependent enhancement (ADE) [19]. Prior studies have shown that non‐neutralizing, cross‐reactive antibodies may facilitate viral entry into Fc gamma receptor‐bearing cells, thereby enhancing viral replication and promoting inflammation [19, 20].

Given this complex epidemiological and immunological landscape, the present study aimed to reassess the circulation of DENV in hospitalized patients during a major dengue outbreak in 2016 at the Central Hospital of Binh Dinh province, central Vietnam [21]. In addition, we sought to investigate the potential serological cross‐reactivity of DENV with other relevant flaviviruses, including JEV, WNV, TBEV, and ZIKV as well as the alphavirus chikungunya (CHIKV).

## 2 Materials and Methods

### 2.1 Ethical Approval Statement

All participants provided signed informed consent for the anonymized use of their blood samples in research, including testing for flaviviruses. For subjects under 18 years of age, consent was obtained from their parents or legal guardians. The study was approved by the Institutional Review Board of the Military Medical University in Hanoi, Vietnam (Approval no. 103MCH/RES/DENV‐GER_V‐D1‐2016), the Binh Dinh Medical College in Gia Lai, Vietnam (Approval no. 466‐QĐ/CĐYT), and the University of Tübingen, Germany (Approval no. 274/2022B02), for the project entitled “Host and Viral Factors Influencing Dengue Severity and Susceptibility.” Authorization for the use of Vietnamese genetic resources in Germany was granted in accordance with the Nagoya Protocol obtained from the Vietnamese Ministry of Natural Resources and Environment (Reference No. 2995/QĐ‐BTNMT). All procedures followed GCP/GCLP guidelines and were in accordance with the ethical standards of the Helsinki Declaration.

### 2.2 Study Population

Samples were collected during the 2016 dengue outbreak in Binh Dinh Central Hospital, spanning March to June in 2016. This retrospective study included 146 patients diagnosed with dengue who were admitted to the provincial Central Hospital. Dengue diagnoses followed the World Health Organisation diagnostic′s criteria [5], as adopted by the Vietnamese Ministry of Health. The inclusion criteria were patients presenting with fever within 7 days of onset, accompanied by at least two clinical signs or symptoms suggestive of dengue (e.g., nausea/vomiting, rash, body aches and pains, tourniquet test positive) and positive for at least one of the indirect diagnostic methods (serological rapid test NS1/IgG/IgM), as recommended and detailed in the WHO guideline 2009 [5]. Patients with bacterial or other viral infections, chronic diseases, or hematological disorders were excluded. A total of 10 mL of whole blood was collected from each participant at the time of admission, comprising samples for routine laboratory diagnostics and for research purposes. Plasma was separated and stored at −70°C until use. Clinical severity data for dengue cases were not available in this study cohort.

### 2.3 Patients Laboratory Assessment

The dengue nonstructural protein 1 (NS1) antigen and anti‐DENV immunoglobulin M (IgM) and G (IgG) antibodies were determined upon hospital admission to support clinical diagnosis. The following laboratory tests were conducted at the admitting hospital at the time of admission: white blood cell count (WBC), red blood cell count (RBC), hemoglobin (Hb), hematocrit (HCT), platelet count (PLT), urea, creatinine, aspartate aminotransferase (AST), alanine aminotransferase (ALT), and high‐sensitivity C‐reactive protein (CRP.hs). Primary and secondary dengue infections were differentiated based on the IgM/IgG optical density (OD) ratio using ELISA, with patient plasma diluted at 1:101. According to WHO guidelines and supported by prior studies [5, 22], an OD ratio greater than 1.2 indicates primary infection, while a ratio below 1.2 suggests secondary infection.

### 2.4 Dengue Serotype Detection by Real‐Time RT‐PCR

DENV serotypes in confirmed dengue cases were identified using the RealStar Dengue Type RT‐PCR kit 1.0 (Altona Diagnostics GmbH, Hamburg, Germany) on a LightCycler480‐II (Roche, Mannheim, Germany), following the manufacturer's instructions. All assays were performed in duplicate. Patients with identified DENV serotypes by RT‐PCR were classified as confirmed dengue cases, whereas those without serotype identification but positive by NS1 and/or IgM/IgG tests were classified as probable dengue cases.

### 2.5 Serological Assays

To investigate cross‐reactivity with other viral hemorrhagic fever‐related pathogens, plasma IgG and IgM antibodies against five flavivirus pathogens (DENV, JEV, ZIKV, WNV, and TBEV) and one alphavirus (CHIKV) were detected using commercial ELISA kits (Euroimmun, Lübeck, Germany). The specific kits utilized were anti‐dengue virus type 1−4 ELISA (IgG/IgM), anti‐JEV ELISA (IgG/IgM), anti‐ZIKV ELISA (IgG/IgM), anti‐WNV ELISA (IgG/IgM), anti‐TBEV ELISA 2.0 (IgG/IgM), and anti‐CHIKV ELISA (IgG/IgM).

All assays were performed in duplicate following the manufacturer′s instructions. Briefly, plasma samples were diluted at a ratio of 1:101 and incubated at 37°C for 60 min. Samples were then incubated sequentially with the conjugate solution for 30 min and the substrate solution for 15 min at room temperature, with three washes performed between each step. The reaction was terminated using a stop solution, and absorbance was measured at wavelengths of 450 and 620 nm with a CLARIOstar microplate reader (BMG Labtech, Ortenberg, Germany). Results were interpreted according to the manufacturer‐defined cut‐off indices provided with each kit.

### 2.6 Amplification and Sequencing of the Dengue Capsid‐Premembrane (CprM)

A representative subset of samples (*n* = 30) from DENV‐1‐positive patients was selected for amplification of the CprM region. cDNA was synthesized from viral RNA using the LunaScript RT‐SuperMix, following the manufacturer′s protocol. The primers for CprM region amplification were those described by Lanciotti et al. [23]. The PCR product from the first round of amplification (PCR‐outer, lengths ~511 bp) were used for phylogenetic analysis. In brief: PCR reactions were performed in 20 μL reaction volume with 3 μL of synthesized cDNA (approx. 5 ng cDNA), 1x buffer (Qiagen GmbH, Hilden, Germany), 0.5 μM of each primer, 200 μM of dNTPs, and 1U (unit) of Taq DNA polymerase (Qiagen GmbH, Hilden, Germany). The thermal cycling parameters consist of an initial denaturation at 94°C for 3 min, followed by 35 cycles of denaturation (30 s at 94°C), annealing (60 s at 55°C), extension (60 s at 72°C), followed by a final extension at 72°C for 10 min. PCR amplicons were stained with SYBR green and visualized on a 1.2% gel electrophoresis.

PCR products were purified using Exo‐SAP‐IT (Applied Biosystems, Beverly, MA, USA) and used for the sequencing reaction using the BigDye Terminator v.1.1 Cycle Sequencing Kit on an ABI 3130XL DNA sequencer (Applied Biosystems, Beverly, MA, USA). Sequencing reactions were done for both strands using forward and reverse primers. The sequences were assembled and checked for nucleotide ambiguities manually using Seqman version 6.1 (DNASTAR, Lasergene, USA). The consensus sequences were verified using National Center for Biotechnology Information (NCBI) BLAST (http://blast.ncbi.nlm.nih.gov/Blast.cgi).

### 2.7 DENV Phylogenetic Analysis

The sequences were aligned using ClustalW in MEGA version 11 [24], with respective reference sequences for DENV‐1: NC001477. The phylogenetic analysis was performed using MEGA. The phylogenetic tree was reconstructed using the maximum likelihood method based on the Kimura 2‐parameter model with 1000 bootstrap iterations. Reference sequences from various geographical regions were obtained from NCBI genotyping tool, with accession numbers provided for each serotype. The sequences generated from this study were submitted to GenBank and were assigned the accession numbers PQ562260–PQ562289 (*n* = 30).

### 2.8 Statistical Analysis

The data were analyzed and visualized using the software R version 4.3.2 (http://www.r-project.org). The patient data were presented as median values (with range) for quantitative variables and as absolute numbers (with percentages) for categorical variables. The normality of the distribution of quantitative variables was tested using the Shapiro–Wilk test. Categorical data were compared using the chi‐square test, while continuous variables were compared using the Student's *t*‐test or Wilcoxon test. Pearson correlation coefficients (as the data were parametric) were computed to evaluate relationships between antibody responses to different viruses. A *p*‐value of < 0.05 is considered statistically significant.

## 3 Results

### 3.1 Demographic and Laboratory Data of Study Participants

All patients were of Kinh ethnicity and residents of Binh Dinh (now Gia Lai) province. Overall, patient ages ranged from 1 to 50 years, with 52% of the study population being female. There was no significant difference in sex distribution between patients with primary and secondary DENV infections. The median age of patients with primary infection (10.5 [1–40]) was significantly lower than that of those with secondary infection (20 [2–50]) (*p* = 0.003) (Table 1).

**Table 1:** Patient characteristics on admission stratified by primary and secondary dengue infections.

	Primary infection (*n* = 52)	Secondary infection (*n* = 94)	*p* value
Age (years)	10.5 (1–40)	20 (2–50)	**0.003**
Sex (count female, %)	28 (54%)	48 (51%)	0.863
NS1 (count positive, %)	26 (50%)	49 (52%)	0.863
WBC (×10⁹/L)	3.3 (1.3–11)	3.55 (1.3–12.4)	0.836
RBC (×10¹²/L)	4.71 (3.24–5.88)	4.95 (2.51–8.5)	0.163
Hb (g/L)	138.5 (105–164)	138 (63–185)	0.798
HCT (%)	41.45 (31.5–51.9)	41.65 (11.3–53.2)	0.699
PLT (×10⁹/L)	75.5 (6–225)	77 (8–186)	0.598
Urea (mmol/L)	3.58 (1.05–13.6)	3.8 (0.34–9.97)	0.608
Creatinine (µmol/L)	79 (27–109)	78 (36–123)	0.644
AST (U/L)	98 (16.6–1127.7)	97.8 (13.9–8112)	0.902
ALT (U/L)	60.55 (4.5–529.3)	57.2 (5–2835)	0.787
CRP.hs (mg/L)	3.55 (0.4–192)	9.15 (0.2–295)	**0.029**
Serotype (DENV‐1) (positive, %)	47 (90%)	78 (83%)	0.391
IgG positivity			
DENV (count %)	36 (69%)	88 (94%)	**< 0.001**
JEV (count %)	44 (85%)	93 (99%)	**0.001**
TBEV (count %)	37 (71%)	90 (96%)	**< 0.001**
WNV (count %)	36 (69%)	89 (95%)	**< 0.001**
ZIKV (count %)	10 (19%)	32 (34%)	0.085
CHIKV (count %)	0 (0%)	5 (5%)	0.161
IgM positivity			
DENV (count %)	38 (73%)	37 (39%)	**< 0.001**
JEV (count %)	29 (56%)	28 (30%)	**0.003**
TBEV (count %)	9 (17%)	5 (5%)	**0.036**
WNV (count %)	13 (25%)	3 (3%)	**< 0.001**
ZIKV (count %)	0 (0%)	0 (0%)	**0.001**
CHIKV (count %)	0 (0%)	3 (3%)	0.553

*Note:* Variables are summarized as median (range) for continuous data and absolute counts (percentages) for categorical data. *p*‐values were determined from the comparison between primary (*n *= 52) and secondary (*n *= 94) dengue infections, using the chi‐square test for categorical variables and the student's *t*‐test/Wilcoxon rank‐sum test for continuous variables. *p*‐value in bold: statistically significant.

Abbreviations: ALT, alanine aminotransferase; AST, aspartate aminotransferase; CHIKV, Chikungunya virus; CRP.hs, high‐sensitivity C‐reactive protein; DENV, dengue virus; Hb, hemoglobin; HCT, hematocrit; IgG, immunoglobulin G; IgM, immunoglobulin M; JEV, Japanese encephalitis virus; NS1, nonstructuralnonstructural protein 1; PLT, platelet count; RBC, red blood cell count; TBEV, Tick‐borne encephalitis virus; WBC, white blood cell count; WNV, West Nile virus; ZIKV, Zika virus.

Laboratory parameters in the study population deviated from normal physiological ranges (Table 1). In our study population, the median WBC counts were low, consistent with viral infection, PLT counts were markedly reduced (median of 75 × 10³/μL), and elevated levels of AST (median of 97 U/L) and ALT (median of 59 U/L) evidenced the liver damage in dengue (Table 1). Additionally, CRP.hs levels were elevated in majority of patients (> 1 mg/L), indicating systemic inflammation or acute infection. When comparing primary and secondary infections, CRP.hs levels were significantly higher in secondary dengue cases (median 9.15 [0.2–295] vs. 3.55 [0.4–192]; *p* = 0.029), suggesting a more pronounced inflammatory response in these patients (Table 1).

### 3.2 DENV Serotyping and Phylogenetic Analysis

A total of 146 samples were subjected to DENV serotype differentiation. All four DENV serotypes were detected, with DENV‐1 being the most prevalent, identified in 86% of cases (*n* = 125/146) (Table 2). DENV‐4 was found in seven patients (5%), while DENV‐2 and DENV‐3 were each detected in only one case (1%). One patient (1%) presented a co‐infection with DENV‐3 and DENV‐4. In 10 patients (7%), the DENV serotype could not be determined (Supporting Information S1: Table S2).

**Table 2:** Distribution of dengue virus serotypes.

DENV‐1 *n* (%)	DENV‐2 *n* (%)	DENV‐3 *n* (%)	DENV‐4 *n* (%)	DENV‐3 and DENV‐4 *n* (%)	Unidentified *n* (%)	Total (*n*)
125 (86)	2 (1%)	1 (1%)	7 (5%)	1 (1%)	10 (7%)	146

Abbreviations: DENV‐1,2,3,4, dengue virus serotype 1,2,3,4; DENV‐3 and DENV‐4, coinfection of dengue virus serotype 3 and dengue virus serotype 4.

**Figure 1:**
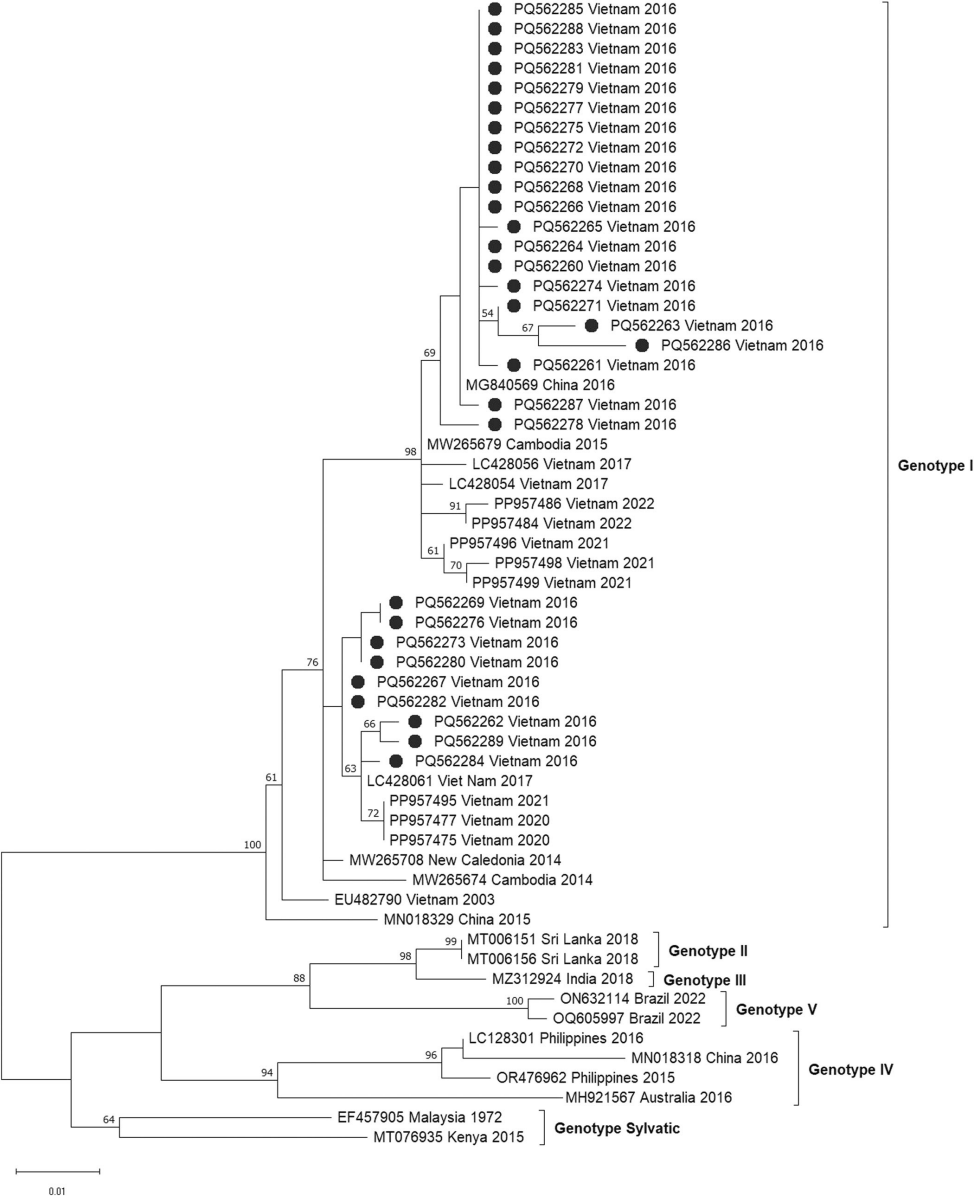
Phylogenetic analysis of dengue virus serotype 1**.** The phylogenetic tree was reconstructed using the maximum likelihood method based on the Kimura 2‐parameter model with 1000 bootstrap iterations. The scale bar indicates a sequence divergence of 0.1. The sequence obtained from this study is highlighted with black circle.

To assess the genetic characteristics of the predominant DENV‐1 strain, partial sequences of the capsid‐premembrane (CprM) gene from 30 DENV‐1‐positive samples were aligned with reference sequences from various geographic regions available in the NCBI database. Phylogenetic analysis revealed that all DENV‐1 strains clustered within genotype I. These sequences showed high similarity to strains previously circulating in Vietnam (2003), Cambodia (2014–2015), China (2016), and New Caledonia (2014) (Figure 1). This suggests a continued circulation and regional persistence of DENV‐1 subtype I in Vietnam, as similar strains were also reported in the country in 2003, 2017, and again between 2020 and 2022 (Figure 1).

### 3.3 Serological Profiles in Study Participants

Serological profiles differed significantly between primary and secondary dengue infections. Patients with secondary infections showed markedly higher IgG seropositivity across multiple flaviviruses, including DENV (94% vs. 69%, *p* < 0.001), JEV (99% vs. 85%, *p* = 0.001), TBEV (96% vs. 71%, *p* < 0.001), and WNV (95% vs. 69%, *p* < 0.001) (Table 1). In contrast, IgM positivity was significantly higher in primary infections for DENV (73% vs. 39%, *p* < 0.001), JEV (56% vs. 30%, *p* = 0.003), TBEV (17% vs. 5%, *p* = 0.036), and WNV (25% vs. 3%, *p* < 0.001), suggesting probable recent infections (Table 1). No IgM positivity for ZIKV was observed in either group. Anti‐CHIKV IgG/IgM positivity also remained low in the study population (Table 1).

Among the 10 samples with unidentified DENV serotypes, five showed anti‐DENV IgM positivity, suggesting recent dengue infections (Supporting Information S1: Table S2). One case (sample ID: BD072) was more consistent with JEV infection, as indicated by the presence of anti‐JEV IgM and the absence of IgM against other tested viruses. Another case (sample ID: BD087) showed probable infection with TBEV, based on the detection of anti‐TBEV IgM alone (Supporting Information S1: Table S2). The remaining samples had broad IgM positivity across multiple flaviviruses, making the etiology unclear.

### 3.4 Correlation of Antibody Responses

**Figure 2:**
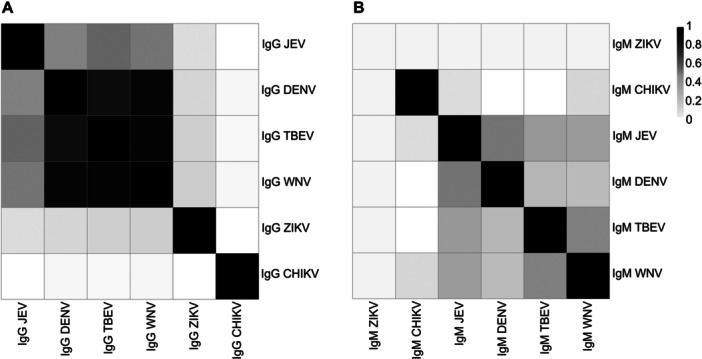
Correlation of IgG (A) and IgM (B) antibody responses. CHIKV, Chikungunya virus; DENV, dengue virus; IgG, immunoglobulin G; IgM, immunoglobulin M; JEV, Japanese encephalitis virus; TBEV, Tick‐borne encephalitis virus; WNV, West Nile virus; ZIKV, Zika virus.

Strong correlations were observed among IgG responses to multiple flaviviruses, indicating substantial serological cross‐reactivity within the study population. IgG against TBEV showed the highest correlation with WNV (*r* = 0.94) and DENV (*r* = 0.86), followed closely by its correlation with JEV (*r* = 0.58) (Figure 2 and Supporting Information S1: Table S1). Similarly, IgG responses to WNV were strongly correlated with DENV (*r* = 0.92) and moderately with JEV (*r* = 0.54), underscoring strong IgG cross‐reactivity among four viruses: DENV, WNV, TBEV, and JEV (Figure 2 and Supporting Information S1: Table S1). In contrast, IgG responses to ZIKV and CHIKV showed weaker correlations with the other tested viruses (*r* < 0.2).

Among IgM responses, no samples tested positive for anti‐ZIKV IgM. However, moderate correlations were noted, especially between JEV and DENV (*r* = 0.50) and between JEV and TBEV (*r* = 0.36), suggesting cross‐reactive IgM responses among flaviviruses (Figure 2 and Supporting Information S1: Table S1). Notably, anti‐WNV IgM was correlated with anti‐TBEV IgM (*r* = 0.48) and anti‐JEV IgM (*r* = 0.35), consistent with the patterns seen in IgG responses, where cross‐reactivity was evident among DENV, WNV, TBEV, and JEV (Figure 2 and Supporting Information S1: Table S1). In contrast, anti‐CHIKV IgM showed minimal or no correlation with other viruses, suggesting distinct or nonoverlapping immune responses.

## 4 Discussion

Our retrospective analysis of the 2016 dengue outbreak in central Vietnam confirms the co‐circulation of all four DENV serotypes during the study period, with DENV‐1 genotype I identified as the predominant strain. Additionally, patients with active dengue infection exhibited strong IgG and IgM cross‐reactivity with TBEV, JEV, and WNV, underscoring the challenges associated with serology‐based diagnostic methods.

The large 2016 dengue outbreak in Binh Dinh Province (now Gia Lai Province since July 2025), which occurred in the same year as Vietnam′s first confirmed ZIKV cases, presents a notable opportunity to investigate arboviral co‐circulation and diagnostic challenges in the region [21, 25]. Most hospitalized patients in our study had secondary dengue infections, with their significantly higher median age supporting a higher likelihood of prior dengue exposure. Secondary infections have been associated with more severe clinical outcomes, characterized by heightened inflammatory responses that contribute to increased vascular permeability, plasma leakage, and multiorgan involvement [19]. In our cohort, secondary infections were associated with elevated levels of high‐sensitivity CRP, a nonspecific but sensitive biomarker of systemic inflammation. Elevated CRP may reflect the intensity of immune activation in dengue infection, which can trigger a cytokine storm characterized by an exaggerated release of pro‐inflammatory cytokines, and is often associated with poor prognosis [26]. Although patients were enrolled within 7 days of fever onset, the exact day of illness onset was not uniformly recorded. This limits the resolution with which IgM/IgG ratio dynamics can be interpreted and precludes accurate assessment of disease severity or correlation with specific clinical manifestations in this cohort. Furthermore, the small sample size and group imbalance constrain the generalizability of our findings, particularly in comparisons involving PLT, HCT, and liver enzymes, which are key indicators in dengue pathophysiology.

DENV‐1 accounted for 86% of all infections in our study. This serotype has been frequently associated with symptomatic dengue and has been implicated in more severe clinical outcomes in both primary and secondary infections [27, 28]. In Vietnam, DENV‐1 and DENV‐2 have been the predominant circulating serotypes over the past decade, with a shift from DENV‐1 to DENV‐2 observed during the 2018–2019 period across the country [29, 30]. While DENV‐2, DENV‐3, and DENV‐4 were also detected in our study, their prevalence was considerably lower. Although DENV‐1 was the predominant serotype identified in this cohort, the detection of DENV‐2, DENV‐3, and DENV‐4, at low levels suggests continued circulation of less prevalent serotypes, which may act as hidden reservoirs for future outbreaks. Previous studies have demonstrated that such serotypes can rapidly displace the dominant serotype, particularly when shifts in population‐level immunity create ecological niches for resurgence [9, 29]. These transitions may be further influenced by ADE, which can exacerbate both viral transmission and disease severity upon secondary infection.

Notably, we identified one case of co‐infection with DENV‐3 and DENV‐4, highlighting the genetic diversity and simultaneous circulation of multiple serotypes in the region. While co‐infections with multiple DENV serotypes were not commonly detected, even isolated occurrences may have significant evolutionary implications, including the potential for genetic recombination or reassortment, which could lead to the emergence of novel viral variants with altered pathogenicity, transmissibility, or immune escape capacity [31]. These findings reinforce the need for high‐resolution genomic surveillance, particularly in hyperendemic regions, where overlapping epidemics and fluctuating serotype dominance increase the complexity of outbreak prediction and control.

To better understand DENV‐1 circulation, we performed phylogenetic analysis using a fragment of the capsid‐premembrane gene. All DENV‐1 sequences belonged to genotype I, a lineage introduced into Vietnam from Thailand in the 1980s–1990s and later from Cambodia in the 2000s [29]. Our sequences clustered with Vietnamese isolates from 2003, 2017, and 2020–2022, indicating sustained local circulation. Although data from southern Vietnam during 2016–2017 are limited, DENV‐1 genotype I was also the dominant strain in a major northern Vietnam outbreak in 2017 and persisted through 2022 [7, 30]. The dominance of a particular DENV strain is difficult to predict, as it can be influenced by various factors, including climatic and ecological conditions, human mobility, population immunity, and vector transmission dynamics [32]. Since shifts in circulating DENV strains can significantly influence outbreak intensity, these complexities underscore the importance of continuous molecular surveillance to strengthen outbreak preparedness and enable timely public health responses. Due to sample limitations, we were unable to perform sequencing for the DENV‐2, DENV‐3, and DENV‐4 cases identified in this study. This limitation restricts our ability to comprehensively characterize the genotypic diversity and evolutionary relationships of all circulating DENV serotypes during the 2016 outbreak.

Vietnam is endemic for multiple flaviviruses, many of which co‐circulate and share overlapping clinical features, making accurate diagnosis particularly challenging during dengue outbreaks [2, 16]. Our study revealed that IgG and IgM antibodies to JEV, WNV, and TBEV were frequently detected in dengue‐confirmed patients with identified DENV serotypes, suggesting that these anti‐JEV/WNV/TBEV serological positives likely reflect cross‐reactivity or limited specificity of the assays rather than true co‐infections or prior exposures to JEV, WNV, or TBEV. The rarity and sporadic reporting of human cases involving these viruses in Vietnam further supports this interpretation [15, 16, 17, 18]. Nonetheless, in the case of JEV, widespread vaccination since 1997 may have contributed to the high IgG seroprevalence observed in our study population, particularly given the median age of overall patients of 19 years [17]. These uncertainties in distinguishing between cross‐reactive IgG responses and true past exposures highlight the need for confirmatory testing, such as virus‐specific neutralization assays, to better clarify the sero‐epidemiological landscape of flaviviruses in the region. The lack of neutralization assays, such as the plaque reduction neutralization test (PRNT), is a limitation of this study, as it restricts our ability to differentiate true past flavivirus exposure from cross‐reactive antibody responses. Future studies should incorporate PRNT or NS1‐based, serotype‐specific ELISAs to enhance the accuracy of seroprevalence estimates and to clarify infection histories, especially in regions with known co‐circulation of multiple flaviviruses.

In contrast, IgM responses demonstrated higher virus specificity, as reflected by lower correlations in seropositivity between different flaviviruses compared to IgG responses. This likely originates from the early‐phase nature of IgM production by naïve B cells, which have not yet undergone somatic hypermutation or affinity maturation, resulting in reduced cross‐reactivity [33, 34]. Particularly, our observation of absent anti‐ZIKV IgM and significantly lower anti‐ZIKV IgG seropositivity in all dengue cases supports the hypothesis that ZIKV elicits less cross‐reactive responses with DENV than other flaviviruses, consistent with previous reports [35]. These findings highlight the diagnostic utility of IgM in identifying recent infections and improving specificity in resource‐limited settings. It is important to consider that co‐infections or prior exposures to different flaviviruses may contribute to broader IgM positivity, potentially affecting diagnostic interpretation. However, this possibility was beyond the scope of this study and warrants further investigation.

Recent studies have highlighted that serological cross‐reactivity among flaviviruses can not only confound diagnosis but also affect clinical outcomes [16, 20]. In individuals with prior flavivirus exposure or vaccination, non‐neutralizing antibodies may contribute to enhanced immune responses during secondary DENV infection, potentially increasing disease severity and hospitalization risk. Emerging evidence further suggests that immune priming from unrelated viral infections, such as SARS‐CoV‐2 may modulate host immune responses during dengue infection, although the exact mechanisms remain incompletely understood [36, 37]. These findings underscore the importance of integrated immunological surveillance in regions where flaviviruses and other viral pathogens co‐circulate.

In our study, we were unable to determine the DENV serotype in 10 patients, and several factors may account for this. One possibility is misdiagnosis, as some patients may have been infected with other flaviviruses such as JEV or TBEV (Supporting Information S1: Table S2), which can elicit cross‐reactive antibodies and result in false‐positive dengue serology [38, 39]. Another explanation is that these patients may have presented during the later stages of dengue infection, when viral RNA levels typically fall below detectable limits, leading to false‐negative PCR results (Supporting Information S1: Table S2). Real‐time RT‐PCR is highly effective for detecting acute DENV infections during the early phase of illness and is considered the gold standard for dengue diagnosis, with optimal sensitivity within the first 2–7 days of symptom onset [40, 41]. In contrast, IgM ELISA played an important role by detecting recent infections that may have been missed by PCR due to declining viremia at the time of sample collection, as IgM antibodies typically begin to rise after Day 4–5 of illness and persist during the convalescent phase [10].

An additional limitation of our study is the extended storage duration of blood samples, which were collected during the 2016 outbreak and stored for several years before being subjected to research analyses. Even under optimal storage conditions, long‐term preservation may lead to partial degradation of viral RNA or reduced antibody stability, which could affect the sensitivity and reliability of both molecular and serological assays. This factor may partly account for the non‐confirmed DENV cases and the complex serological profiles observed in a subset of patients. Additional diagnostic approaches, such as PCR targeting highly conserved genomic regions or viral genome sequencing, may be beneficial for confirming the etiology of these cases and enhancing overall diagnostic accuracy [42]. These findings suggest that strengthening surveillance systems, particularly through the ongoing identification of circulating flaviviruses, will be essential for improving outbreak prediction, guiding public health responses, and mitigating the impact of future hemorrhagic fever epidemics.

## 5 Conclusion

This study highlights the predominance of DENV‐1 genotype I and the high proportion of secondary infections during the 2016 dengue outbreak in central Vietnam, with elevated high‐sensitive CRP levels indicating a heightened inflammatory response in secondary infections. Extensive IgG cross‐reactivity among flaviviruses, particularly DENV, JEV, WNV, and TBEV, complicated serological diagnosis, while IgM responses remained more virus‐specific. These findings underscore the need for integrated molecular and serological surveillance strategies to improve diagnostic accuracy and support effective outbreak management in flavivirus‐endemic regions.

## Author Contributions

T.P.V., N.L.T. designed, supervised the study, and contributed to the study materials and assays. L.H.S. and T.P.V. were involved in the conceptualization and contributed to the study materials. D.D.A., D.H.L. performed the experimental procedures, statistical analysis, and validation of the results. D.T.H.D., N.T.H., N.H.L., and N.T.H.N. recruited the patients and contributed to the investigation materials for sampling procedures. D.A.A. and D.H.L. wrote the first draft. D.D.A., D.H.L, T.P.V., and P.G.K. reviewed the first draft. All authors have read and approved the manuscript.

## Conflicts of Interest

The authors declare no conflicts of interest.

## Supporting information


**Supplementary Table1:** Correlation Matrix of IgG and IgM antibody responses. **Supplementary Table2:** Serological Profile of Samples without Identified DENV Serotype.

## Data Availability

The data that support the findings of this study are openly available in GenBank at https://www.ncbi.nlm.nih.gov/genbank/, reference number PQ562260‐PQ562289. All data are available in the main text or the supplementary materials.
