# Supplementary material for: Dominance of DENV‐1 and Flavivirus Serological Cross‐Reactivity During the 2016 Dengue Outbreak in Vietnam
Source: J Med Virol. 2025 Aug 21;97(8):e70569. doi: 10.1002/jmv.70569 (PMC12369675; doi:10.1002/jmv.70569)
Supplement: Supplementary file 1 — Supplementary Table1: Correlation Matrix of IgG and IgM antibody responses. Supplementary Table2: Serological Profile of Samples without Identified DENV Serotype. [file JMV-97-e70569-s001.docx]

**Supplementary Table1: Correlation Matrix of IgG and IgM antibody responses**

| **IgG** | | | | | | |
| --- | --- | --- | --- | --- | --- | --- |
|  | JEV | ZIKV | CHIKV | TBEV | WNV | DENV |
| JEV | 1 | 0.16 | 0.05 | 0.58 | 0.54 | 0.53 |
| ZIKV | 0.16 | 1 | 0.05 | 0.2 | 0.22 | 0.18 |
| CHIKV | 0.05 | 0.05 | 1 | 0.07 | 0.08 | 0.08 |
| TBEV | 0.58 | 0.2 | 0.07 | 1 | 0.94 | 0.86 |
| WNV | 0.54 | 0.22 | 0.08 | 0.94 | 1 | 0.92 |
| DENV | 0.53 | 0.18 | 0.08 | 0.86 | 0.92 | 1 |
| **IgM** | | | | | | |
|  | JEV | ZIKV | CHIKV | TBEV | WNV | DENV |
| JEV | 1 | NA | 0.08 | 0.36 | 0.35 | 0.5 |
| ZIKV | NA | NA | NA | NA | NA | NA |
| CHIKV | 0.08 | NA | 1 | -0.05 | 0.1 | -0.05 |
| TBEV | 0.36 | NA | -0.05 | 1 | 0.48 | 0.22 |
| WNV | 0.35 | NA | 0.1 | 0.48 | 1 | 0.21 |
| DENV | 0.5 | NA | -0.05 | 0.22 | 0.21 | 1 |

^Abbreviations: IgG: immunoglobulin G; IgM: immunoglobulin M; DENV: Dengue virus; JEV: Japanese encephalitis virus; ZIKV: Zika virus; CHIKV: Chikungunya virus; TBEV: Tick-borne encephalitis virus; WNV: West Nile virus. NA: not applicable.^

**Supplementary Table2: Serological Profile of Samples without Identified DENV Serotype**

|  | | | **^IgM^** | | | | | | **^IgG^** | | | | | | **^Probable^** |
| --- | --- | --- | --- | --- | --- | --- | --- | --- | --- | --- | --- | --- | --- | --- | --- |
| **^ID^** | **^Serotype^** | **^NS1^** | **^DENV^** | **^JEV^** | **^TBEV^** | **^WNV^** | **^ZIKV^** | **^CHIKV^** | **^DENV^** | **^JEV^** | **^TBEV^** | **^WNV^** | **^ZIKV^** | **^CHIKV^** |  |
| ^BD058^ | ^Unidentified^ | ^-^ | ^Positive^ | ^Positive^ | ^Positive^ | ^Positive^ | ^-^ | ^-^ | ^Positive^ | ^Positive^ | ^Positive^ | ^Positive^ | ^-^ | ^-^ | ^DENV^ |
| ^BD076^ | ^Unidentified^ | ^Positive^ | ^Positive^ | ^-^ | ^-^ | ^-^ | ^-^ | ^-^ | ^Positive^ | ^Positive^ | ^Positive^ | ^Positive^ | ^Positive^ | ^-^ | ^DENV^ |
| ^BD108^ | ^Unidentified^ | ^-^ | ^Positive^ | ^Positive^ | ^-^ | ^-^ | ^-^ | ^-^ | ^Positive^ | ^Positive^ | ^Positive^ | ^Positive^ | ^Positive^ | ^-^ | ^DENV^ |
| ^BD115^ | ^Unidentified^ | ^-^ | ^Positive^ | ^Positive^ | ^-^ | ^-^ | ^-^ | ^-^ | ^Positive^ | ^Positive^ | ^Positive^ | ^Positive^ | ^Positive^ | ^-^ | ^DENV^ |
| ^BD134^ | ^Unidentified^ | ^-^ | ^Positive^ | ^Positive^ | ^Positive^ | ^Positive^ | ^-^ | ^-^ | ^Positive^ | ^Positive^ | ^Positive^ | ^Positive^ | ^-^ | ^-^ | ^DENV^ |
| ^BD072^ | ^Unidentified^ | ^-^ | ^-^ | ^Positive^ | ^-^ | ^-^ | ^-^ | ^-^ | ^Positive^ | ^Positive^ | ^Positive^ | ^Positive^ | ^Positive^ | ^-^ | ^JEV^ |
| ^BD114^ | ^Unidentified^ | ^-^ | ^-^ | ^Positive^ | ^-^ | ^-^ | ^-^ | ^Positive^ | ^Positive^ | ^Positive^ | ^Positive^ | ^Positive^ | ^Positive^ | ^Positive^ | ^Uncertainty^ |
| ^BD087^ | ^Unidentified^ | ^-^ | ^-^ | ^-^ | ^Positive^ | ^-^ | ^-^ | ^-^ | ^Positive^ | ^Positive^ | ^Positive^ | ^Positive^ | ^Positive^ | ^-^ | ^TBEV^ |
| ^BD118^ | ^Unidentified^ | ^-^ | ^-^ | ^-^ | ^-^ | ^-^ | ^-^ | ^-^ | ^Positive^ | ^Positive^ | ^Positive^ | ^Positive^ | ^Positive^ | ^-^ | ^Uncertainty^ |
| ^BD128^ | ^Unidentified^ | ^-^ | ^-^ | ^-^ | ^-^ | ^-^ | ^-^ | ^-^ | ^Positive^ | ^Positive^ | ^Positive^ | ^Positive^ | ^-^ | ^Positive^ | ^Uncertainty^ |

^Abbreviations: IgG: immunoglobulin G; IgM: immunoglobulin M. “-“: Negative. DENV: Dengue virus; JEV: Japanese encephalitis virus; ZIKV: Zika virus; CHIKV: Chikungunya virus; TBEV: Tick-borne encephalitis virus; WNV: West Nile virus.^
